# Hidden in plain sight: abdominopelvic pain unveiling a Spigelian hernia containing ovary and fallopian tube

**DOI:** 10.1093/jscr/rjae548

**Published:** 2025-01-09

**Authors:** Joel Ketner, Jason M Lizalek, Elizabeth Maginot, Bennett B Berning

**Affiliations:** College of Medicine, University of Nebraska Medical Center, Omaha, NE 68198, United States; Division of Acute Care Surgery, Department of Surgery, University of Nebraska Medical Center, Omaha, NE 68198, United States; Division of Acute Care Surgery, Department of Surgery, University of Nebraska Medical Center, Omaha, NE 68198, United States; Division of Acute Care Surgery, Department of Surgery, University of Nebraska Medical Center, Omaha, NE 68198, United States

**Keywords:** spigelian hernia, ovary, fallopian tube, laparoscopic, case report

## Abstract

Spigelian hernias are rare clinical entities; vague symptomatology and unreliable clinical examination ensure difficult diagnosis. Computed tomography (CT) is critical for accurate diagnosis. Surgical repair is mandated given the high risk of visceral organ incarceration. Few cases have reported herniation of the ovary or fallopian tube. A 76-year-old female presented with bilateral lower quadrant pain associated with tenderness and no palpable bulge. A CT scan identified a right lower quadrant Spigelian hernia containing the right ovary and fallopian tube. A laparoscopic transabdominal preperitoneal repair with mesh was performed. Intraoperative evaluation showed a congested ovary. The patient was discharged postoperative Day 1. Spigelian hernias can involve the small intestine, greater omentum, or colon, while cases involving the gynecologic organs are rare. CT is key to diagnosis. A minimally invasive surgical approach should be considered given its potential benefits of decreased wound complications and its diagnostic and therapeutic utility.

## Introduction

Spigelian hernias are rare ventral hernias characterized by a defect in the aponeuroses of the transversus abdominis and internal oblique near the semilunar line. Located 6 cm cephalad to the anterior superior iliac spines, the Spigelian hernia belt is the anatomic location most associated with this anatomic defect. Incidence is reported to be ~1–2% of abdominal wall hernias and those patients at highest risk are female patients in the sixth and seventh decade of life [1]. Spigelian hernias are more likely to become incarcerated compared with other types of hernias; therefore, clinical suspicion is necessary as ~30% of patients can present without clinical examination findings despite symptoms [1, 2]. The most likely incarcerated organs include the small intestine, greater omentum, and sigmoid colon [1]. There have been few reports of incarcerated ovary and/or fallopian tube within the literature [3, 4].

## Case report

**Figure 1:**
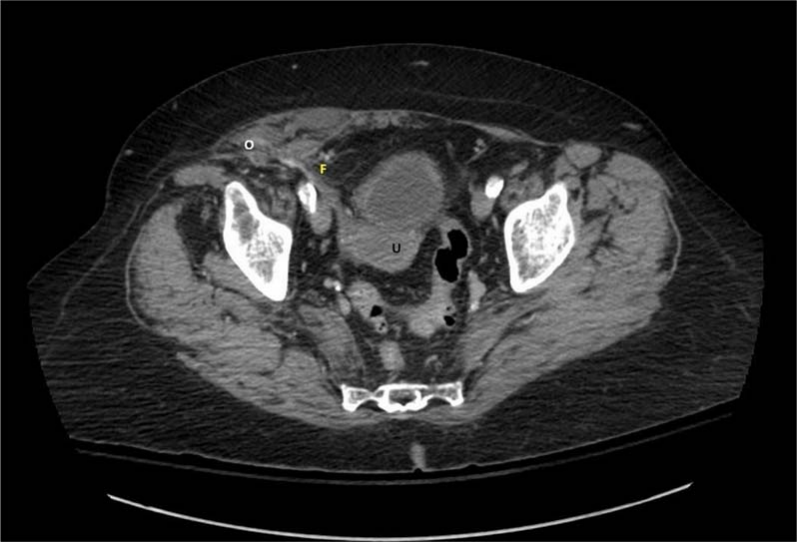
Axial view of contrast-enhanced computed tomography of the abdomen and pelvis demonstrating the right fallopian tube (F) attached to the uterus (U) extending into the Spigelian hernia defect containing the right ovary (O).

**Figure 2:**
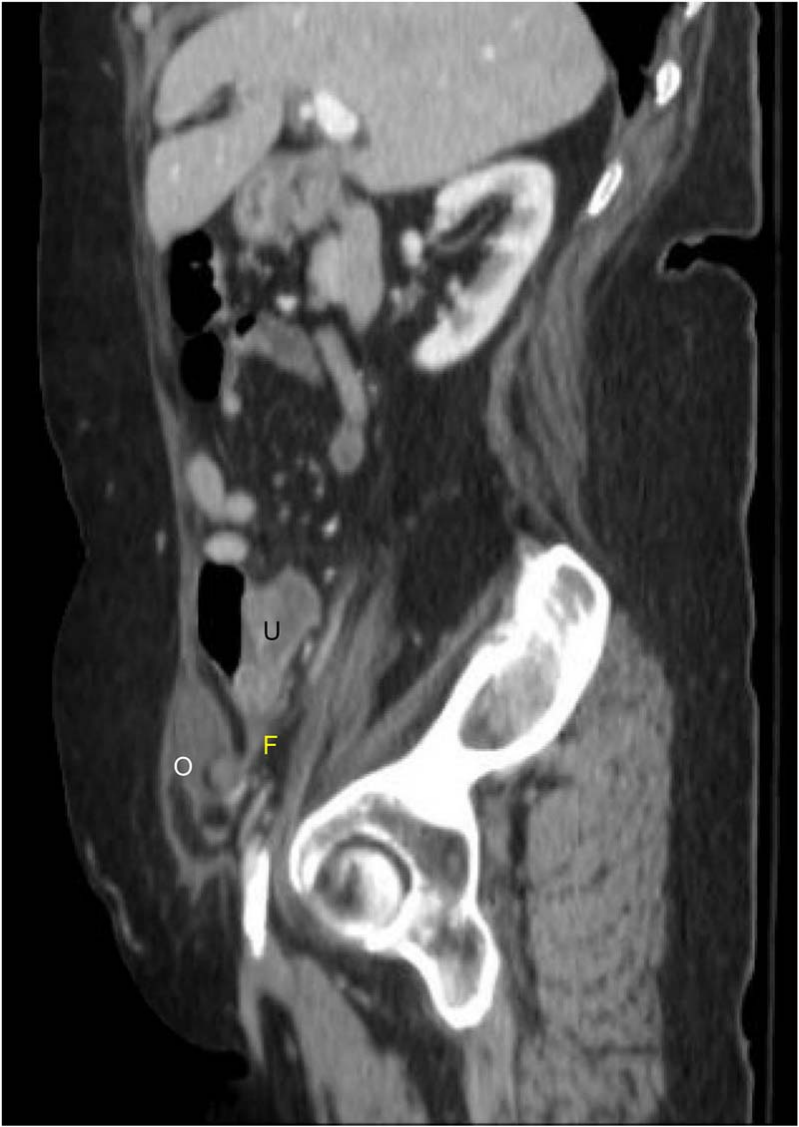
Sagittal view of contrast-enhanced computed tomography of the abdomen and pelvis demonstrating the right fallopian tube (F) attached to the uterus (U) extending into the Spigelian hernia defect containing the right ovary (O).

A 76-year-old Caucasian female presented to the emergency department for evaluation of 3 days of progressively worsening abdominal pain. The pain was characterized as an ache, originated in the right lower quadrant and radiated to the bilateral upper quadrants. Past medical history was significant for obesity (BMI 31 kg/m^2^), chronic obstructive pulmonary disease, gastroesophageal reflux disease, hypertension, hypothyroidism, heart failure with preserved ejection fraction, atrial fibrillation, and scleroderma. Pertinent surgical history included a dual-chamber pacemaker and tubal ligation. She was anticoagulated with apixaban. Physical examination revealed right lower quadrant tenderness to palpation and rebound tenderness without a palpable abdominal bulge. Contrast-enhanced computed tomography of the abdomen and pelvis did not clearly demonstrate the appendix, but rather a ventral hernia in the lower right quadrant. The fascial defect was lateral to the rectus abdominis, superior to the inguinal ligament, and inferior to the arcuate line. The defect measured 3.6 × 2.0 cm and involved the transversus abdominis and internal oblique muscle aponeuroses while the external oblique remained intact, consistent with Spigelian hernia (Fig. 1). There was an oblong structure extending parauterine into the hernia sac with a small amount of free fluid suggestive of the right ovary and fallopian tube (Fig. 2). Considering these findings and appropriate cessation of anticoagulation, the patient was taken for diagnostic laparoscopy and transabdominal preperitoneal hernia repair.

Surgery was performed under general anesthesia in the supine position. Pneumoperitoneum was obtained using the Veress technique. Laparoscopic ports were placed. The hernia defect was identified in the lower right quadrant. There were no herniated visceral contents; however, the right ovary was near the defect and appeared congested. The defect measured 2 × 4 cm located at the semilunar line. The preperitoneal plane was developed to accommodate appropriate mesh overlap and the hernia sac was reduced. The fascial defect was reapproximated with 2–0 auto-locking suture. A 0 braided, absorbable suture was placed through the center of a 15 × 7.5 cm macroporous polypropylene mesh (Parietene™), and it was introduced into the peritoneal cavity. The mesh was positioned over the center of the closed fascial defect and the braided suture was grasped with a suture passer, delivered transfascial, and secured to approximate the mesh against the abdominal wall. The mesh was secured superior and lateral using an absorbable automatic tacking device. A total of 10 ml of fibrin glue (Tisseel®, Baxter) was used to fix the mesh medial, and inferior to ensure no iatrogenic injury to the epigastric and external iliac vessels, respectively. The peritoneum was reapproximated with 2–0 auto-locking suture. The postoperative course was uneventful, and the patient was discharged on postoperative Day 1. There was no evidence of recurrence at 1 month follow-up.

## Discussion

The reported case highlights the utility of computed tomography to accurately diagnose Spigelian hernia anatomy when history and clinical exam findings are vague. It also recognizes the need for the surgeon to maintain a broad differential diagnosis for a patient presenting with right lower quadrant abdominal pain. This case demonstrates the feasibility of laparoscopic repair of a Spigelian hernia in the acute care setting. Previous reports of Spigelian hernia containing intra-abdominal viscera, particularly the ovary and fallopian tube, have described only an open approach to repair [3, 4]. There is no official recommendation from the European Hernia Society or Americas Hernia Society on specific surgical method, open or minimally invasive. However, both organizations acknowledge the possibility of decreased wound complications in addition to the diagnostic and therapeutic potential of laparoscopic approach in patients without palpable abnormality [5]. In this case, a laparoscopic transabdominal approach not only allowed for assessment of the herniated ovary and fallopian tube, but also preperitoneal repair with mesh. While a multitude of clinical pathology can present with acute-onset right lower quadrant abdominopelvic pain, the general surgeon should remain vigilant for Spigelian hernia due to the high risk of incarceration and need for prompt surgical intervention.

## Data Availability

Not applicable.
